# An improved method for identifying functionally linked proteins using phylogenetic profiles

**DOI:** 10.1186/1471-2105-8-S4-S7

**Published:** 2007-05-22

**Authors:** Shawn Cokus, Sayaka Mizutani, Matteo Pellegrini

**Affiliations:** 1 Department of Molecular, Cell, and Developmental Biology, University of California, Los Angeles, CA 90095-1606, USA

## Abstract

**Background:**

Phylogenetic profiles record the occurrence of homologs of genes across fully sequenced organisms. Proteins with similar profiles are typically components of protein complexes or metabolic pathways. Various existing methods measure similarity between two profiles and, hence, the likelihood that the two proteins co-evolve. Some methods ignore phylogenetic relationships between organisms while others account for such with metrics that explicitly model the likelihood of two proteins co-evolving on a tree. The latter methods more sensitively detect co-evolving proteins, but at a significant computational cost. Here we propose a novel heuristic to improve phylogenetic profile analysis that accounts for phylogenetic relationships between genomes in a computationally efficient fashion. We first order the genomes within profiles and then enumerate runs of consecutive matches and accurately compute the probability of observing these. We hypothesize that profiles with many runs are more likely to involve functionally related proteins than profiles in which all the matches are concentrated in one interval of the tree.

**Results:**

We compared our approach to various previously published methods that both ignore and incorporate the underlying phylogeny between organisms. To evaluate performance, we compare the functional similarity of rank-ordered lists of protein pairs that share similar phylogenetic profiles by assessing significance of overlap in their Gene Ontology annotations. Accounting for runs in phylogenetic profile matches improves our ability to identify functionally related pairs of proteins. Furthermore, the networks that result from our approach tend to have smaller clusters of co-evolving proteins than networks computed using previous approaches and are thus more useful for inferring functional relationships. Finally, we report that our approach is orders of magnitude more computationally efficient than full tree-based methods.

**Conclusion:**

We have developed an improved method for analyzing phylogenetic profiles. The method allows us to more accurately and efficiently infer functional relationships between proteins based on these profiles than other published approaches. As the number of fully sequenced genomes increases, it becomes more important to account for evolutionary relationships among organisms in comparative analyses. Our approach, therefore, serves as an important example of how these relationships may be accounted for in an efficient manner.

## Background

To date, about 400 bacterial genomes have been fully sequenced. Although these sequences provide us with a wealth of information, the functions of the products of many of the genes they contain have yet to be characterized. Development of methodologies that can predict their function is an important goal for bioinformatics. The most widely used methods for protein function prediction are based on the detection of homologies via sequence alignments. These approaches are often insufficient, however, as many proteins have no functionally characterized homologs. Moreover, it is not possible to completely define the function of an isolated protein as function depends intimately on contextual information such as interactions, pathways, and cellular localizations.

**Figure 1:**
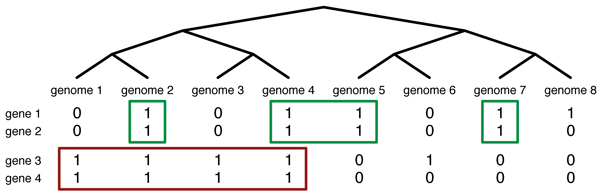
**Phylogenetic profiles**. We show hypothetical phylogenetic profiles for four genes. Genes 1 and 2 have four common 1's ("matches") in three runs while genes 3 and 4 have four matches in a single run. We hypothesize that genes 1 and 2 are more likely to be truly co-evolving while genes 3 and 4 are likely to be just lineage-specific.

Functional characterization of proteins using phylogenetic profiles has emerged as an important technique during the past few years [1]. A phylogenetic profile is a {0,1}-vector that is assigned to each protein within a genome and whose elements indicate the absence and presence of homologs of the protein in other genomes (see Figure 1). The underlying assumption of methods that utilize these profiles is that proteins that function together tend to co-occur across organisms. Thus, clusters of proteins with similar profiles correspond to pathways and complexes, and participation in such a cluster may be used as evidence that an uncharacterized protein shares this function.

Various metrics have been used to quantify similarity between two phylogenetic profiles, including Hamming distance [1], probability of matches using the hypergeometric distribution [2], and mutual information [3]. However, these metrics do not consider the underlying phylogeny of the genomes in the profile. As Figure 1 suggests, there is ample reason to believe that accounting for phylogeny should improve our ability to detect truly co-evolving genes (genes 1 and 2) from those that are merely present in a subset of related genomes (genes 3 and 4).

In contrast to these approaches, another class of methods has been developed to account for genome phylogeny when scoring profile similarities [4-7]. These approaches reconstruct phylogenetic trees and estimate gene loss and gain events at branch points to identify proteins that appear to co-evolve. These methods are more complex and computationally expensive than those of the previous paragraph. For this reason, significant computational resources are required to apply these methods to all-versus-all comparisons of proteins in bacterial genomes. As a result, we set out to develop a heuristic approach that is computationally more efficient than existing full tree-based methods and yet partially accounts for phylogenetic relationships among organisms when scoring profile pairs.

Our approach involves two components. The first computes the probability of two profiles having a certain number of matches using an extension of the hypergeometric distribution that accounts for the number of proteins in each genome. The underlying assumption is that protein pairs that possess profiles with more matches are more likely to co-evolve. The second component partially accounts for the underlying phylogeny between organisms by first ordering the genomes within the profile by their similarity. We then compute runs of consecutive matched homologs in phylogenetic profiles to distinguish between conservation across disparate species versus conservation of occurrences within clusters of related organisms. Each component is described by readily computable formulae, and the two components are easy to mathematically combine to yield a single score that two particular profiles are significantly similar.

We compare our method to several previously published approaches for phylogenetic profile comparison: computing the probability of matches between two profiles using the hypergeometric distribution [2], measuring the similarity of profiles using mutual information [3], using a reduced set of genomes in the profile to eliminate closely related organisms [8], estimating profile similarity while accounting for genome occupancy [9], and estimating similarity by using likelihood ratios to compare two maximum-likelihood models of gene evolution using a full phylogenetic tree [7]. We compare these approaches by measuring how often proteins in significantly similar profile pairs share the same Gene Ontology (GO) terms [10]. We demonstrate that our method compares favorably to these other approaches in terms of both performance and computational efficiency.

In conclusion, we have developed an efficient method to account for genome phylogenies when computing phylogenetic profile similarities. We show that this approach improves our ability to reconstruct various pathways and complexes, including, as an example, the subunits of nitrate reductases. In the future, we plan to incorporate this new methodology into the Prolinks database [11].

## Results

We began with previously computed phylogenetic profiles constructed from 214 genomes [12]. These profiles had been computed for each reference organism using BLAST [13] to define the presence and absence of homologs across the genomes. In this paper, we focus our analysis on the approximately 4,200 genes of the genome of *Escherichia coli K12 *as they have the most comprehensive annotations and therefore allow us to more accurately assess the performance of methods. However, there is no reason to expect that the results are specific to *E. coli*, and we therefore expect the method to perform well if any of the fully sequenced genomes are used as reference.

We computed the similarity of phylogenetic profiles using pairwise scores for each possible pair of distinct proteins in *E. coli*. We compared several different metrics for computing the significance of the similarity between two given profiles. The first is the *p*-value for the number of matches (common 1's) between two profiles being large as computed from the appropriate hypergeometric distribution [2]. The underlying assumption is that more matches between two profiles correspond to an increased likelihood that two proteins co-evolved. (Under a null hypothesis that the two genes are not co-evolving and assuming that the 1's in the profile of each gene are uniformly randomly distributed, the null hypothesis translates to independence of the two profiles and the number of matches takes on one of the standard discrete probability distributions of statistics, a hypergeometric distribution.) This approach assumes that all positions in the profile are equally likely to be populated by 1's (an assumption relaxed starting with the third method described below) and that the order of the positions in the profile does not matter (an assumption relaxed in the fifth and sixth methods below). Hence, phylogenetic relationships between the organisms that make up the profile are not considered in this first method. The second approach utilizes mutual information to estimate profile similarity [3] and is very similar in practice to the first method.

A slightly more sophisticated approach involves a weighted hypergeometric distribution to estimate the number of matches [9]. This approach accounts for the different size of each genome by assigning a probability, or *weight*, of occupancy at each position in the profile. (For example, if a genome contains 42% of the genes in the reference genome, then its weight is 0.42.) Genomes highly similar to the reference genome have weights near 1 while those more distant from it have lower weights. Weighted *p*-values reduce to unweighted *p*-values when all the weights are the same (which is not the case here). As with the previous two methods, however, this does not account for phylogenetic relationships between organisms.

The fourth approach begins to consider phylogeny by utilizing only a reduced set of genomes in the profiles in order to remove highly similar organisms that might confound the comparisons [8]. For this, instead of the full 214-dimensional profile vectors, we used only 157 organisms by selecting a single representative genome from groups of highly related taxa (for details, see Methods).

The fifth and final approach we compare against estimates co-evolution between two proteins based on gains and losses on a full phylogenetic tree [7]. In this approach, two models of evolution are compared, one model having the two proteins co-evolve and the other having them evolve separately. For each pair of profiles, maximum likelihood point estimates of several parameters describing gene loss and gain rates are determined and then the likelihood ratio of the two models is used as the statistic for the gene pair. A detailed description of this sophisticated approach is beyond the scope of the present paper and may be found in the original publication. Due to the high computational cost of applying this approach to our data using the software provided by the authors, we computed likelihood ratios for only a random sampling of 100,000 GO-benchmarkable pairs of proteins (= ~2.8% of benchmarkable pairs = ~1.1% of all pairs) rather than for all 8,817,900 pairs (of which 3,525,840 are benchmarkable by GO) in all the other methods. With approximately 5–15 CPU seconds required per pair on a contemporary PC, a complete all-versus-all run of this method requires more than 1 CPU year whereas a full run of the other methods (including the new method presented here) can be done in under 2.5 CPU minutes each. Hence, this last method is many orders of magnitude more expensive to compute than any of the others.

We compare the five approaches above to a sixth developed here that combines a weighted hypergeometric *p*-value with a penalty that is a *p*-value for the number of "runs" being unusually small. The weighted hypergeometric *p*-value is the same as that described above (and note that it incorporates the size of each genome when estimating the overlap between two profiles). The second scoring component is the probability of having the observed number of runs or fewer in the overlap vector. A *run *is defined as a maximal non-empty string of consecutive occupancy matches between two profiles. An example is provided in Figure 1. Genes 1 and 2 share four organisms distributed over three runs, while genes 3 and 4 also have four matches but only in a single run. We hypothesize that given the underlying phylogenetic tree shown in Figure 1, the matches between genes 1 and 2 are less likely to occur by chance than the ones between genes 3 and 4. The reason is that more events are required to account for the pattern seen between genes 1 and 2, and, hence, these two genes are more likely to be truly co-evolving and thus related functionally.

The number of runs depends on the ordering of genomes within the phylogenetic profiles. We attempted to establish an ordering that reflects the evolutionary relationships among the organisms. To this end, we first constructed a genome-genome distance matrix based on the phylogenetic profile data itself. If one encodes the phylogenetic profile data as a {0,1}-matrix whose rows are the proteins and whose columns are the genomes, then the *genome phylogenetic profiles *are the columns. Given their genome phylogenetic profiles, we use Jaccard dissimilarity (i.e., percentage of disagreeing positions among positions where at least one gene has a 1) to measure distance between two genomes.

To identify a good ordering of genomes, we perform hierarchical clustering of them using the genome-genome distance matrix of the previous paragraph. This process generates a dendrogram that represents the evolutionary relationships among organisms [14]. However, naïve hierarchical clustering is only topological and there remains ambiguity about the ordering of genomes because at each non-leaf the left and right subtrees may be exchanged or "swivelled." To optimize swivels, we use dynamic programming to minimize the sum of squared distances between adjacent genomes across the leaves of the dendrogram [15]. (Note that brute-force search is infeasible as the number of swivellings is exponential in the number of genomes and is large even for small numbers of genomes.)

Having computed a good ordering of genomes, we next compute the probability of obtaining an equal number of or fewer runs than the number actually observed. Details are summarized in the Methods section and fully explained in Additional File 1. In our final model, we combine the weighted hypergeometric *p*-value with our *p*-value for the number of runs by dividing the former by the latter (hence, on a logarithmic scale, the latter is subtracted from the former). This simple combination was found to work well in practice. As described in Additional File 1, our methods permit the incorporation of numerous additional terms into this combination, but we feel this basic two-term model is simple, achieves good performance, and has intuitive appeal.

The relative performance of methods is evaluated using GO annotations [10]. GO is organized into three separate ontologies: cellular compartment, biological process, and molecular function. We use the first two ontologies to evaluate protein pairs since similarities in molecular function are largely determined by conventional sequence alignment-based methods. Each ontology is organized as a directed acyclic graph (DAG). Very general terms at the top are parent terms for more specific terms deeper down in the ontology. The genes themselves are among the leaves of the DAG. In each of the ontologies, any two proteins always share at least one term (if only the root) as long as both have at least one annotation to the ontology. However, proteins with closely related functions will share at least one very specific term while those with only distantly related function will share only more general terms. To determine the functional similarity between two proteins, we therefore compute the probability that the highest specificity of their common terms is as high as it is, as described in detail in Methods.

**Figure 2:**
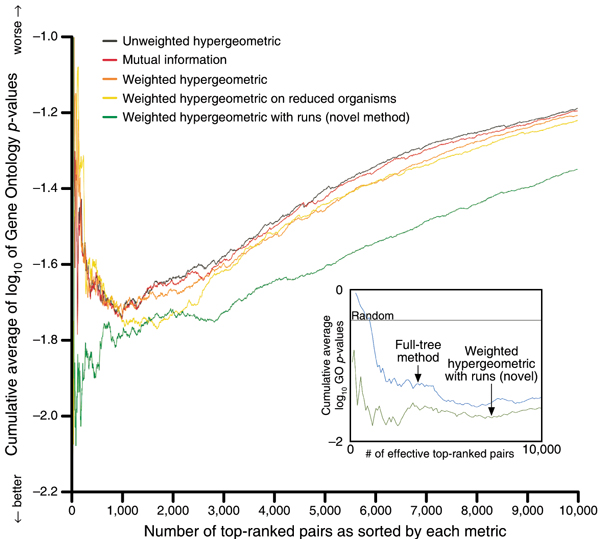
**Benchmarking six methods for pairwise comparison of phylogenetic profiles**. We compare six methods for ranking pairs of phylogenetic profiles. The first (black) uses the unweighted hypergeometric distribution for the probability of the observed or a greater number of matches between two profiles. The second (red) ranks by *mutual information*, the entropy of the first profile plus the entropy of the second profile minus the entropy of the joint profile viewed one genome at a time [3]. The third (orange) uses the weighted hypergeometric distribution that considers the occupancy of each genome across all genes. The fourth (yellow) is the same as the third but on a reduced set of organisms. The fifth (green) combines the weighted hypergeometric *p*-value and a *p*-value for the observed or a smaller number of runs in the observed matches. Methods are benchmarked against the GO cellular localization and biological process ontologies. The *GO p-value *for each pair of proteins is the probability for the genes of that pair to share a GO term at least as specific as their most specific shared term, and we compute the cumulative average log_10 _GO *p*-value for top pairs as ranked by each metric. Introducing runs into the calculations improves results by tending to yield more significant GO *p*-values. The inset compares the fifth method (green) to a full tree-based method (blue). Due to the computational difficulty of evaluating Pagel's method, we only compared it to our novel method on a random subset of 100,000 benchmarkable pairs. Each such sampled pair represents approximately 35 pairs in a full all-versus-all run. The average log_10 _GO *p*-value over all benchmarkable pairs is approximately -0.40 and is shown in the inset (but lies above the top of the main plot).

To test the performance of a metric, we compute the cumulative average log_10 _GO *p*-value as we move down the list of protein pairs in the metric's rank order. (These cumulative averages rank different metrics in the same way as would be obtained from the regularized incomplete gamma functions taken by products of independent uniform [0,1] random variables to produce single *p*-values from collections of multiple *p*-values.) As shown in Figure 2, we see that our method incorporating runs outperforms both the weighted hypergeometric and the unweighted hypergeometric methods as well as the much more computationally expensive full tree-based method. In particular, we note that the top 1,000 pairs are significantly more similar in their GO terms when we account for runs.

**Figure 3:**
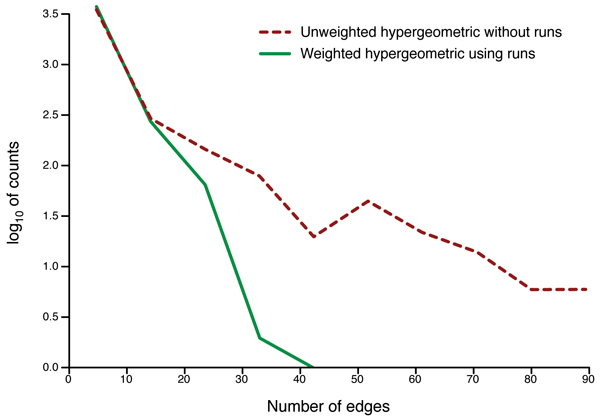
**Network degree distribution**. We show the edge distribution for two networks, one built using the top 5,000 pairs according to the unweighted hypergeometric metric without runs and one from our runs-employing two-term model. We see that the unweighted hypergeometric network contains many more edges of high degree. In particular, nodes with more than 40 edges are almost completely absent from the runs network while being abundant in the unweighted hypergeometric network. This suggests that the runs-informed network contains smaller and more interpretable clusters.

To further analyze the effect of including runs in our analysis, we compared the top 5,000 pairs generated by the pure unweighted hypergeometric and by the runs-using methods. First, we note that of 5,000 pairs, 3,458 are in common while 1,542 are not, indicating that the resulting networks are significantly different. More importantly, we observe that the distribution of edges in the two networks is quite different. In Figure 3, we see that while the pure hypergeometric network contains many nodes with 40 or more edges, the runs-informed network has almost none. This is consistent with the pure hypergeometric network containing very large clusters of linked proteins while the runs-informed network is broken down into smaller clusters. This is significant because large clusters are not very useful for functional studies since they bring together proteins with a broad range of functions. In contrast, small clusters can contain proteins with well-defined functional relationships.

**Figure 4:**
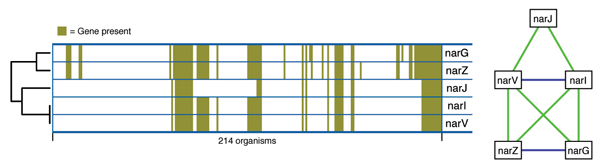
**Nitrate reductase subnetwork**. We show a small cluster that is present in the runs-informed network and absent in the unweighted hypergeometric network that does not use runs. The phylogenetic profiles of the corresponding genes are shown in (*a*). Significant edges are shown in (*b*) with blue edges being identified by both methods while green edges belong only to the runs-informed network. We note that the only elements of this network detected by the non-runs-using method are two highly homologous nitrate reductase complex subunits; the other members are less homologous and are missed by it. Even though these genes occur in relatively few genomes, those in which they occur are widely scattered and form many runs in the profiles, leading to their correct inclusion in the runs-informed network.

As an example, we focus on a small cluster of proteins shown in Figure 4 that are found in the runs-informed network but not in the pure hypergeometric network. The cluster contains many subunits of two nitrate reductases: narG and narZ are the alpha subunits of nitrate reductase I and II, respectively; narI and narV are the gamma subunits; and narJ is the delta subunit. We see that while the number of genomes that contain all these proteins is relatively small (thus explaining why this cluster does not appear in the pure hypergeometric analysis), they are scattered positionally throughout the profiles and form many runs (hence their inclusion in the runs-informed network). It is clear that these proteins do indeed belong together as they are subunits of a protein complex that catalyzes the reduction of nitrate to ammonia. We also note that the cluster contains two distinct complexes, nitrate reductase I and II, that are highly homologous. This inability to separate homologous or parallel complexes is one limitation of phylogenetic profile analyses that we have noted in the past [16].

**Figure 5:**
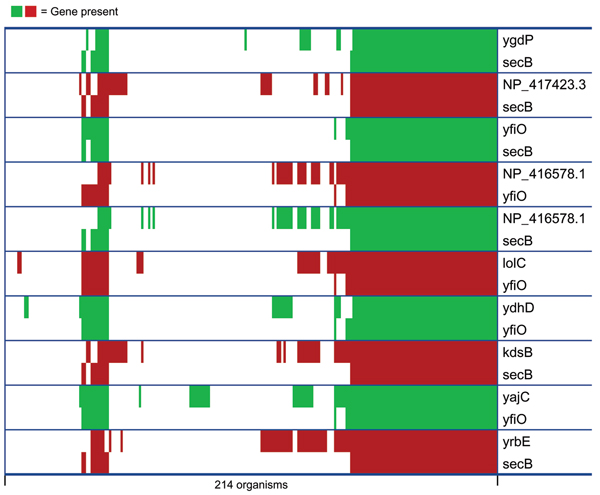
**Profile pairs preferred by the unweighted hypergeometric metric without runs**. Shown are the top 10 pairs of profiles that score highly in unweighted hypergeometric *p*-value but poorly in the runs-informed metric as determined by smallest ratios of unweighted hypergeometric *p*-value without runs to our runs-using score (taking both on a linear and not on a logarithmic scale). Not surprisingly, the matches between these profiles are concentrated in few runs. We find that the protein pairs here are not closely related functionally according to our snapshot of GO, and so they are likely false positives for the runs-oblivious unweighted hypergeometric model.

A final example of the different performance of the pure hypergeometric metric versus the runs-based approach is shown in Figure 5. Here we have selected 10 pairs of proteins whose profiles are significantly similar according to the pure hypergeometric criterion but not according to the runs-based method. As expected, we see that most of the matches between these pairs are clustered in just a few runs, thus explaining the difference in significance as computed by the two methods. Further, most of these pairs do not appear to be biologically relevant. Many of the pairs involve secB, a molecular chaperone involved in protein export. This protein is paired with the nucleotide hydrolase ygdP, the CMP-3-deoxy-D-manno-octulosonate transferase kdsB, and several hypothetical proteins. Although we cannot know for sure, it does not seem likely that most of these proteins share a functional relationship with secB. As a result, this example illustrates how pairs of proteins with few runs are less likely to be functionally related.

## Discussion

There are three general classes of metrics that may be used to compare two binary phylogenetic profiles. The first class is insensitive to the underlying phylogeny of organisms and treats each position in the profile completely independent of the others. Members of this class of metrics are highly represented in the literature [1-3] and are very straightforward to implement. However, these metrics suffer significantly from their underlying assumptions, especially as the number of genomes in the profiles increases.

The second class of metrics assumes that the underlying organismal phylogenetic tree is known and takes advantage of this prior knowledge when computing profile similarities. Several examples of this kind of approach have been described in the literature in the past few years [4-7]. Although these approaches have been shown to outperform the first type of metric, they do so at considerable computational expense. Furthermore, they depend critically on the prior tree, which is only suggestive of historical fact (due to incomplete information, implementational approximations to reconstruction, horizontal transfer events, and other problems).

The third class of metrics is represented by our heuristic approach that considers only an ordering of genomes and not a full phylogenetic tree. We have shown that this approach is superior to the first type of metric as one might expect and can even outperform the second class of approaches. Another advantage is that our approach is intermediate in conceptual complexity between the first and second class of metrics. Most significantly, and in contrast to the full tree-based methods, the computational requirements of our approach are modest, and therefore it is suitable for large-scale applications in which hundreds of millions of profile pairs need to be compared. As a result, we believe that the approach described here represents an appealing solution to the problem of phylogenetic profile comparison.

## Conclusion

Genomic sequencing is advancing at a remarkable pace as new technologies supplement traditional approaches [17]. The number of sequenced organisms, now standing at about 400, will undoubtedly reach into the thousands in a short time. This deluge of data presents us with several challenges and opportunities. One challenge is to understand the function and interrelationships among the proteins coded within these genomes. The opportunity is to develop a new generation of computational approaches that allow us to accomplish this without using expensive and time-consuming experimental techniques.

Phylogenetic profiles are one of the approaches aimed at this goal. Phylogenetic profiles now represent a fairly mature approach for determining protein function when traditional homology-based techniques fail. Nonetheless, current implementations of the technique are either overly simple and do not account for organism phylogenies or overly complex and require very significant computational resources to implement on a large scale.

Here we have presented a third type of approach that measures similarity between phylogenetic profiles given only an ordering of organisms and without knowledge of the tree. Although the "correct" ordering among organisms within a profile cannot be known exactly, we have shown that easily constructed orderings allow one to significantly improve the performance of phylogenetic profiles compared to naïve approaches and reach a performance that is superior even to that of full tree-based approaches. As the number of available genomes increases, using approaches such as this one will be critical for effective use of phylogenetic profiles and will bring us closer to the goal of developing efficient and accurate methodologies for inferring protein functions from sequence data alone.

## Methods

### Phylogenetic profiles

Profiles for 214 bacterial and archaeal genomes were obtained from the Tavazoie lab at the Lewis-Sigler Institute for Integrative Genomics. Specifically, data at the Web site [18] accompanying Slonim et al. [12] was used. The weights of the genomes vary considerably, with ~25% each below 0.23, between 0.23 and 0.34, between 0.34 and 0.45, and above 0.45. Nearly 9% of genomes have a weight as extreme as below 0.15 or above 0.85.

### Weighted hypergeometric and weighted runs *p*-values

Full derivation and discussion of the computation of the primary *p*-values used here (among others) is contained in Additional File 1. While the derivation may be difficult to follow for those unused to the combinatorial language of generating functions, the ultimate mechanics of how the *p*-values are computed for up to a few hundred genomes are quite easy, are summarized here, and do not require understanding of the derivation. Notation here agrees with that of Supplemental File 1. Let *w*_*i *_for *i *in 1..*n *be the weight of genome *i*, which is the fraction in (0,1) of the 4,200 reference genes contained in genome *i*.

For weighted hypergeometric *p*-values, start with a 1-by-1-by-1 cubical array *P' *of real floating-point values consisting of a single element +1.0. For each *i *in 1..*n *in turn, replace the array with the entrywise sum of four arrays: (1) the current array with every entry multiplied by (1 - *w*_*i*_)^2 ^and padded by a 1-entry-thick slab of +0.0's on the back, bottom, and right; (2) the current array with every entry multiplied by (1 - *w*_*i*_) *w*_*i *_and padded by a 1-entry-thick slab of +0.0's on the back, top, and right; (3) the current array with every entry multiplied by *w*_*i *_(1 - *w*_*i*_) and padded by a 1-entry-thick slab of +0.0's on the front, bottom, and right; and (4) the current array with every entry multiplied by *w*_i_^2 ^and padded by a 1-entry-thick slab of +0.0's on the front, top, and left.

The probability in our statistical null hypothesis model of no co-evolution of a pair of genes that the number of genomes that have the first gene is some number *a *≥ 0, the number of genomes that have the second gene is some number *b *≥ 0, and the number of genomes that have both genes is some number *c *≥ 0 is the value of the unique entry of *P' *that is simultaneously (*a*+1)^th ^from the front, (*b*+1)^th ^from the top, and (*c*+1)^th ^from the left. The *p*-value, then, that the number of genomes with both genes is at least as large as *c *given *a *and *b *is

Pr⁡(c≥observed|a,b)=Pr⁡(c≥observed,a,b)Pr⁡(a,b)=∑c'=cnP'[a+1,b+1,c'+1]∑c'=0nP'[a+1,b+1,c'+1].
 MathType@MTEF@5@5@+=feaafiart1ev1aaatCvAUfKttLearuWrP9MDH5MBPbIqV92AaeXatLxBI9gBaebbnrfifHhDYfgasaacH8akY=wiFfYdH8Gipec8Eeeu0xXdbba9frFj0=OqFfea0dXdd9vqai=hGuQ8kuc9pgc9s8qqaq=dirpe0xb9q8qiLsFr0=vr0=vr0dc8meaabaqaciaacaGaaeqabaqabeGadaaakeaacyGGqbaucqGGYbGCcqGGOaakcqWGJbWycqGHLjYScqqGVbWBcqqGIbGycqqGZbWCcqqGLbqzcqqGYbGCcqqG2bGDcqqGLbqzcqqGKbazcqGG8baFcqWGHbqycqGGSaalcqWGIbGycqGGPaqkcqGH9aqpdaWcaaqaaiGbccfaqjabckhaYjabcIcaOiabdogaJjabgwMiZkabb+gaVjabbkgaIjabbohaZjabbwgaLjabbkhaYjabbAha2jabbwgaLjabbsgaKjabcYcaSiabdggaHjabcYcaSiabdkgaIjabcMcaPaqaaiGbccfaqjabckhaYjabcIcaOiabdggaHjabcYcaSiabdkgaIjabcMcaPaaacqGH9aqpdaWcaaqaamaaqahabaGaemiuaaLaei4jaCIaei4waSLaemyyaeMaey4kaSIaeGymaeJaeiilaWIaemOyaiMaey4kaSIaeGymaeJaeiilaWIaem4yamMaei4jaCIaey4kaSIaeGymaeJaeiyxa0faleaacqWGJbWycqGGNaWjcqGH9aqpcqWGJbWyaeaacqWGUbGBa0GaeyyeIuoaaOqaamaaqahabaGaemiuaaLaei4jaCIaei4waSLaemyyaeMaey4kaSIaeGymaeJaeiilaWIaemOyaiMaey4kaSIaeGymaeJaeiilaWIaem4yamMaei4jaCIaey4kaSIaeGymaeJaeiyxa0faleaacqWGJbWycqGGNaWjcqGH9aqpcqaIWaamaeaacqWGUbGBa0GaeyyeIuoaaaGccqGGUaGlaaa@9637@

It is useful to post-process *P' *in a single final pass so that *P' *[*a *+ 1, *b *+ 1, *c *+ 1] is directly the desired *p*-value. With this, scoring of a gene pair reduces to a single array access. An implementation created for this work took ~3.5 CPU seconds on a contemporary PC to calculate the array for the *n *= 214 case needed; note that the array only needs to be computed once per all-pairs run.

For weighted runs *p*-values, start with two 1-by-1 rectangular arrays: *Q" *with +1.0 and *Z" *with +0.0. For each *i *in 1..*n *in turn, simultaneously update *Q" *and *Z" *as follows: replace *Q" *with the entrywise sum of the two current arrays after multiplying each element by 1 - *w*_i_^2 ^and padding by a single row and column of +0.0's on the bottom and right, and replace *Z" *by the following: (1) take the entrywise sum of the current *Q" *after padding by a single row and column of +0.0's on the top and left with the current *Z" *after padding by a single row and column of +0.0's on the top and right, then (2) multiply every entry by *w*_i_^2^. Take the entrywise sum of the final *Q" *and *Z" *arrays to obtain array *P"*.

The probability under our null hypothesis that the number of genomes that have both genes is some number *c *≥ 0 and the number of runs is some number *t *≥ 0 is the value of the unique entry of *P" *that is simultaneously (*c*+1)^th ^from the top and (*t*+1)^th ^from the left. The *p*-value, then, that the number of runs is no more than *t *given *c *is

Pr⁡(t≤observed|c)=Pr⁡(t≤observed,c)Pr⁡(c)=∑t'=0tP''[c+1,t'+1]∑t'=0⌈n/2⌉P''[c+1,t'+1].
 MathType@MTEF@5@5@+=feaafiart1ev1aaatCvAUfKttLearuWrP9MDH5MBPbIqV92AaeXatLxBI9gBaebbnrfifHhDYfgasaacH8akY=wiFfYdH8Gipec8Eeeu0xXdbba9frFj0=OqFfea0dXdd9vqai=hGuQ8kuc9pgc9s8qqaq=dirpe0xb9q8qiLsFr0=vr0=vr0dc8meaabaqaciaacaGaaeqabaqabeGadaaakeaacyGGqbaucqGGYbGCcqGGOaakcqWG0baDcqGHKjYOcqqGVbWBcqqGIbGycqqGZbWCcqqGLbqzcqqGYbGCcqqG2bGDcqqGLbqzcqqGKbazcqGG8baFcqWGJbWycqGGPaqkcqGH9aqpdaWcaaqaaiGbccfaqjabckhaYjabcIcaOiabdsha0jabgsMiJkabb+gaVjabbkgaIjabbohaZjabbwgaLjabbkhaYjabbAha2jabbwgaLjabbsgaKjabcYcaSiabdogaJjabcMcaPaqaaiGbccfaqjabckhaYjabcIcaOiabdogaJjabcMcaPaaacqGH9aqpdaWcaaqaamaaqahabaGaemiuaaLaei4jaCIaei4jaCIaei4waSLaem4yamMaey4kaSIaeGymaeJaeiilaWIaemiDaqNaei4jaCIaey4kaSIaeGymaeJaeiyxa0faleaacqWG0baDcqGGNaWjcqGH9aqpcqaIWaamaeaacqWG0baDa0GaeyyeIuoaaOqaamaaqahabaGaemiuaaLaei4jaCIaei4jaCIaei4waSLaem4yamMaey4kaSIaeGymaeJaeiilaWIaemiDaqNaei4jaCIaey4kaSIaeGymaeJaeiyxa0faleaacqWG0baDcqGGNaWjcqGH9aqpcqaIWaamaeaadaWbdaqaaiabd6gaUjabc+caViabikdaYaGaayP74laawMp+aaqdcqGHris5aaaakiabc6caUaaa@90B4@

Again, it is useful to post-process *P" *in a single final pass so that *P" *[*c *+ 1, *t *+ 1] is directly the desired *p*-value so that scoring of a gene pair reduces to a single array access. An implementation created for this work took ~0.016 CPU seconds on a contemporary PC to calculate the array for the *n *= 214 case needed; note again that the array needs to be computed only once per all-pairs run.

If *H *is the weighted hypergeometric *p*-value for a given pair of genes and *R *is the weighted runs *p*-value for the same pair of genes, then we score the pair of genes as *H*/*R *or, on a logarithmic scale, log_10 _*H *- log_10 _*R *= (1) log_10 _*H *+ (-1) log_10 _*R*. The choice of linear combination coefficients (1, -1) was made for its simplicity, good performance, and intuitive appeal after investigation of several members of a family of models. These other models were linear combinations of logs of *p*-values selected among those *p*-values made possible by Supplemental File 1. Linear combination coefficients were trained by robust (iteratively reweighted) linear regression with logs of GO *p*-values as targets on randomly selected small numbers of a variety of interesting gene pairs. We found the intuitively appealing two-term combination with simple coefficients (1, -1) presented in the bulk of this article to be a good simplicity-performance tradeoff, although other models are easily investigated given our framework.

### GO *p*-values

EMBL GOA 18 *E. coli K12 *GO annotations [19] and a 08-07-2005 version of the base GO in OBO format [20] were downloaded. GenBank NP_/YP_-style identifiers from the original profiles were mapped to NCBI GI numbers with Batch Entrez [21], and then *i*ProClass [22] associations in either direction were used to push these to UniProt accessions/identifiers, which were finally matched to the gene labels used in the EMBL GOA file. In this way, 3,013 reference genes of the original 4,200 were mapped into GO.

We restrict to the cellular component ("C") and biological process ("P") ontologies; molecular function ("F") annotations are discarded. Call the *size *of a GO term the number of mapped genes annotated to it directly or indirectly in the GO DAG. Small terms are specific, while large terms are general. Terms of size zero are discarded. To benchmark the strength of association of two genes that have at least one direct or indirect term in common – a *benchmarkable pair *– we use as a statistic the smallest size of all direct and indirect terms they have in common.

This statistic is converted to a *p*-value via a precomputed table of its distribution over all benchmarkable pairs. Specifically, we take as *p*-value the fraction of benchmarkable pairs whose statistic is as small or smaller than observed in the current pair of genes. In other words, the GO *p*-value is the probability that a randomly chosen benchmarkable pair of genes has a common term at least as specific as the most specific term common to the current pair of genes.

### Genome order

As briefly discussed above, the order of genomes is important because the number of runs generally changes as organisms are permuted. To begin determining the order we used, a genome-by-genome distance matrix was constructed from the genome profiles and Jaccard dissimilarity, which is the percentage of disagreeing positions among positions where at least one gene has a 1. Hierarchical clustering with complete linkage to obtain a topological rooted proper binary tree was next performed with *Mathematica*'s Statistics'ClusterAnalysis'DirectAgglomerate[] function (taking ~0.003 CPU seconds on a contemporary PC for the needed *n *= 214 case). A small custom program briefly described below whose algorithm was derived before the publication of Bar-Joseph et al. [15] was used to find the best swivelling of left and right subtrees at every non-leaf so as to minimize the *cost *of the swivelling, which we took to be the sum of the squares of the Jaccard dissimilarities of pairwise adjacent leaves. Additional Files 2 and 3 illustrate the effectiveness of this optimization. The resulting order of leaves was retained for use in the comparisons of all profile pairs, and (for the new method presented here) the tree was otherwise forgotten. Optimization of swivels for the needed *n *= 214 case took ~0.155 CPU seconds on a contemporary PC.

In our case, there are exactly four optimal swivellings. Half of these are obtained from the other half by rigidly flipping the entire tree over (i.e., reflection in a vertical mirror, or simultaneous exchange of left and right subtree at every non-leaf). This symmetry does not affect the number of runs. The other freedom in our case is a transposition of two adjacent organisms, *Tropheryma whipplei TW08/27 *and *Tropheryma whipplei str. Twist*. For the sake of completeness, we chose the order placing *Nanoarchaeum equitans *as leftmost leaf and *TW08/27 *to the left of *Twist*.

Dynamic programming is used to find the optimal swivellings. Denote by *l*(*x*) and *r*(*x*) the left and right child, respectively, of node *x*, or *x *itself if *x *is a leaf. Let **L**(*x*) be the leaves of the subtree rooted at node *x*. For every (*x*, {*a*,*d*}) where *x *is a node and *a *is in **L**(*l*(*x*)) and *d *is in **L**(*r*(*x*)), we keep track of the lowest cost *C*(*x*, {*a*,*d*}) among all swivellings of the subtree rooted at *x *that place *a *as the leftmost leaf and *d *as the rightmost leaf. Write Δ(*b*, *c*) for the additive cost for having leaf node *b *adjacent to leaf node *c *(which we took to be the square of their Jaccard dissimilarity). Then *C*(*x*, {*x*,*x*}) = 0 for every leaf *x*, and we have the following simple recurrence relation for non-leaves *x*:

C(x,{a,d})=min⁡(C(l(x),{a,b})+Δ(b,c)+C(r(x),{c,d})  |   b∈{L(l(l(x)))if a∈L(r(l(x)))L(r(l(x)))otherwise} and   c∈{L(l(r(x)))if d∈L(r(r(x)))L(r(r(x)))otherwise}).
 MathType@MTEF@5@5@+=feaafiart1ev1aaatCvAUfKttLearuWrP9MDH5MBPbIqV92AaeXatLxBI9gBaebbnrfifHhDYfgasaacH8akY=wiFfYdH8Gipec8Eeeu0xXdbba9frFj0=OqFfea0dXdd9vqai=hGuQ8kuc9pgc9s8qqaq=dirpe0xb9q8qiLsFr0=vr0=vr0dc8meaabaqaciaacaGaaeqabaqabeGadaaakeaacqWGdbWqcqGGOaakcqWG4baEcqGGSaalcqGG7bWEcqWGHbqycqGGSaalcqWGKbazcqGG9bqFcqGGPaqkcqGH9aqpcyGGTbqBcqGGPbqAcqGGUbGBdaqadaabaeqabaGaem4qamKaeiikaGIaemiBaWMaeiikaGIaemiEaGNaeiykaKIaeiilaWIaei4EaSNaemyyaeMaeiilaWIaemOyaiMaeiyFa0NaeiykaKIaey4kaSIaeuiLdqKaeiikaGIaemOyaiMaeiilaWIaem4yamMaeiykaKIaey4kaSIaem4qamKaeiikaGIaemOCaiNaeiikaGIaemiEaGNaeiykaKIaeiilaWIaei4EaSNaem4yamMaeiilaWIaemizaqMaeiyFa0NaeiykaKIaeeiiaaIaeeiiaaYaaqqaaeaafaqabeGacaaabaaabaaabaaabaaaaaGaay5bSdaabaGaeeiiaaIaeeiiaaIaeeiiaaIaemOyaiMaeyicI48aaiWaaeaafaqaaeGacaaabaacbeGae8htaWKaeiikaGIaemiBaWMaeiikaGIaemiBaWMaeiikaGIaemiEaGNaeiykaKIaeiykaKIaeiykaKcabaGaeeyAaKMaeeOzayMaeeiiaaIaemyyaeMaeyicI4Sae8htaWKaeiikaGIaemOCaiNaeiikaGIaemiBaWMaeiikaGIaemiEaGNaeiykaKIaeiykaKIaeiykaKcabaGae8htaWKaeiikaGIaemOCaiNaeiikaGIaemiBaWMaeiikaGIaemiEaGNaeiykaKIaeiykaKIaeiykaKcabaGaee4Ba8MaeeiDaqNaeeiAaGMaeeyzauMaeeOCaiNaee4DaCNaeeyAaKMaee4CamNaeeyzaugaaaGaay5Eaiaaw2haaiabbccaGiabbggaHjabb6gaUjabbsgaKbqaaiabbccaGiabbccaGiabbccaGiabdogaJjabgIGiopaacmaabaqbaeaabiGaaaqaaiab=XeamjabcIcaOiabdYgaSjabcIcaOiabdkhaYjabcIcaOiabdIha4jabcMcaPiabcMcaPiabcMcaPaqaaiabbMgaPjabbAgaMjabbccaGiabdsgaKjabgIGiolab=XeamjabcIcaOiabdkhaYjabcIcaOiabdkhaYjabcIcaOiabdIha4jabcMcaPiabcMcaPiabcMcaPaqaaiab=XeamjabcIcaOiabdkhaYjabcIcaOiabdkhaYjabcIcaOiabdIha4jabcMcaPiabcMcaPiabcMcaPaqaaiabb+gaVjabbsha0jabbIgaOjabbwgaLjabbkhaYjabbEha3jabbMgaPjabbohaZjabbwgaLbaaaiaawUhacaGL9baaaaGaayjkaiaawMcaaiabc6caUaaa@E0B9@

(Once the root, leftmost leaf, and rightmost leaf are fixed, an optimal swivelling has to place some node *b *as the rightmost leaf of the left subtree and some node *c *as the leftmost leaf of the right subtree and use an optimal swivelling for each of these two subtrees.) It is easy to compute all values of *C*(*x*, {·,·}) inductively on *x *from the bottom of the tree toward the root, finishing *x *for the left and right child of a node before beginning that node. The optimal cost for swivelling the whole tree is min(*C*(root, {*a*,*d*}) | *a *in **L**(*l*(root)) and *d *in **L**(*r*(root))).

Actual optimal swivellings themselves are found with a backtracking phase similar to that used with other optimization problems solved with dynamic programming, such as sequence alignment. Backtracking information (i.e., the argument values attaining the various minimums) can either be recorded during the first pass of computation or be recomputed as needed during backtracking. To illustrate, the left subtree of the root is swivelled if and only if the argmin *a *for the root is in **L**(*r*(*l*(root))).

### Reduced genome profiles

To test whether removal of similar genomes from the phylogenetic profiles improves performance, we developed a procedure for selecting from the total set of genomes a subset that does not contain close relatives. We first determined groups of highly related organisms. These groups were selected by successively undoing cluster joins in the Jaccard dendrogram in order of most-to-least similar. We chose to stop clustering when all four *E. coli *genomes were grouped together; there were many groups of reasonable size and content at this point.

Computing the mean Jaccard distance from each organism in the group to the other organisms in the group and selecting the one with the smallest mean allowed us to select a representative organism from each group. If multiple organisms satisfied this criterion, the group was temporarily enlarged to include the leaves of the subtree rooted at the group's lowest common ancestor, and mean distances were computed from each organism in the original group to organisms in the enlarged group. If there still was no unique minimum mean distance, then we further temporarily enlarged the group, going up the tree until there was a unique minimum. Except for deletion of organisms, organism order was otherwise kept unchanged.

### Full tree-based method

BayesTraits executables and the bms_runner script were downloaded from the Web site of the Pagel lab [23]. Optimization of a rate-of-gains parameter dependent on the particular phylogenetic profiles used is required, and for this bms_runner requires "true positive" and "true negative" gene pairs. The 3,261 gene pairs with GO *p*-value below 0.001 were taken as true positives, and a random subset of 3,261 pairs from the 3,174,504 benchmarkable pairs with GO *p*-value of 0.1 and above were taken as true negatives. The tree used is that already described under "Genome order" above (with swivelling irrelevant for this method).

Thirty-seven training runs at different values of the parameter between 0 and 9.5 including a single unrestricted run were performed at a cost of approximately one-half CPU day per parameter value on contemporary PCs. Specificity-sensitivity plots were made from scratch as the script's summary output for this was found to be unreliable, and parameter value 0.01 was selected as best.

As the computational costs of a complete all-versus-all run of this method exceeded the resources available to us (due to its requirement of more than 1 CPU year), we scored only a random subset of 100,000 pairs from only the 3,525,840 GO-benchmarkable pairs. This required approximately 1 CPU week.

## Competing interests

The authors declare that they have no competing interests.

## Authors' contributions

SC developed the mathematical formalisms and computed the *p*-values. SM and MP analyzed the results and identified examples. MP and SC wrote the manuscript.

## Supplementary Material

Additional file 1 **Derivation and calculation of primary *p*-values**. This four-page PDF document contains a detailed derivation and discussion of the calculation of the primary *p*-values used in this work, including the weighted hypergeometric *p*-values and weighted runs *p*-values, among others not used in the main article. Click here for file

Additional file 2 **Distance matrix before and after optimal swivelling**. This one-page PDF file shows the hierarchically-clustered-by-complete-linkage genome-genome Jaccard dissimilarity distance matrix before (left) and after (right) optimal swivelling. The improved visual appearance of the swivelled distance matrix is apparent. The effect can be even more dramatic when optimal swivelling is applied to heatmaps of, e.g., microarray expression data. Click here for file

Additional file 3 **Reduction in the number of runs per gene after optimal swivelling**. This one-page PDF file shows the cumulative number of genes as the number of runs in the gene's profile is slowly raised. It is apparent that optimal swivelling tends to reduce the number of runs in a gene's profile. Thus, the organism order derived from optimal swivelling captures the organisms' underlying phylogeny better than the order derived from hierarchical clustering without optimal swivelling (which, in turn, does much better than a random ordering, suggesting that runs can indeed capture phylogenetic information). Click here for file
