# Supplementary material for: An improved method for identifying functionally linked proteins using phylogenetic profiles
Source: BMC Bioinformatics. 2007 May 22;8(Suppl 4):S7. doi: 10.1186/1471-2105-8-S4-S7 (PMC1892086; doi:10.1186/1471-2105-8-S4-S7)
Supplement: Additional file 1 — Derivation and calculation of primary p-values. This four-page PDF document contains a detailed derivation and discussion of the calculation of the primary p-values used in this work, including the weighted hypergeometric p-values and weighted runs p-values, among others not used in the main article. [file 1471-2105-8-S4-S7-S1.pdf]

**Supplemental Methods for *An Improved Method for Identifying Functionally-Linked Proteins Using Phylogenetic Profiles* by Shawn Cokus, Sayaka Mizutani, and Matteo Pellegrini**

### Derivation of $p$-values

Denote by $n \geq 1$ the number of genomes and focus on a pair of genes. Let $a_i$, $b_i$, $c_i \in \{0, 1\}$ be 1 iff genome $i \in 1..n$ has the first gene, second gene, and both genes, respectively. When considering runs, it is useful to have a notional "0th genome" with $a_0 := b_0 := c_0 := 0$. Let $r_i$, $s_i \in \{0, 1\}$ be 1 iff genome $i \in 1..n$ begins a run (i.e., genome $i$ has a 1 and genome $i-1$ has a 0) in the first and second genes, respectively. Finally, let $t_i \in \{0, 1\}$ be 1 iff genome $i \in 1..n$ begins a run in the *both-genes profile* that is 1 exactly at the genomes that have both genes. We take $r_0 := s_0 := t_0 := 0$.

Our statistical null hypothesis model of no co-evolution of genes is as follows. The events "genome $i$ contains gene $j$" are mutually independent over all pairs of genomes and genes. The probability that a given gene is present in genome $i \in 1..n$ is taken to be the fraction of all genes that genome $i$ contains, which we record as the *weight* $w_i \in (0, 1)$. A genome's weight is near 1 when it is closely related to the reference genome and near 0 when it is very distant.

Let $k \in 0..n$ be given and let random variables $A_k$, $B_k$, and $C_k$ taking values in $0..k$ be the number of genomes that have the first gene, second gene, and both genes, respectively, on restriction to genomes $0..k$. Let random variables $R_k$, $S_k$, and $T_k$ taking values in $0..\lceil k/2 \rceil$ be the number of runs in the first gene profile, second gene profile, and both-genes profile on restriction to genomes $0..k$. To obtain the conditional distribution of $C_n$ given $A_n$ and $B_n$ (the "weighted hypergeometric" distribution), the conditional distribution of $T_n$ given $C_n$ (the "weighted runs" distribution), and many other conditional distributions, it suffices to find the joint distribution of $A_n$, $B_n$, $C_n$, $R_n$, $S_n$, and $T_n$.

The combinatorial language of generating functions is extremely well-suited for statistical models of phylogenetic profiles, including our present one. Probability distributions are represented as multivariate polynomials with real coefficients in $[0, 1]$. While a multivariate polynomial is nothing more than an alternate representation of a multi-dimensional array of numbers, natural mathematical operations upon them (especially polynomial multiplication) directly correspond to features of the statistical models. Further, dynamic programming schemes for efficient computation of actual probabilities are often immediately apparent when generating functions are expressed in particular forms. Therefore, we desire the coefficients of the multivariate polynomial $P_n$ in the six variables $\boldsymbol{a}$, $\boldsymbol{b}$, $\boldsymbol{c}$, $\boldsymbol{r}$, $\boldsymbol{s}$, and $\boldsymbol{t}$, where

$$P_k := \sum_{a=0}^{k}\sum_{b=0}^{k}\sum_{c=0}^{k}\sum_{r=0}^{\lceil k/2\rceil}\sum_{s=0}^{\lceil k/2\rceil}\sum_{t=0}^{\lceil k/2\rceil} \Pr\begin{pmatrix} A_k = a, & B_k = b, & C_k = c, \\ R_k = r, & S_k = s, & T_k = t \end{pmatrix} \cdot \boldsymbol{a}^a \boldsymbol{b}^b \boldsymbol{c}^c \boldsymbol{r}^r \boldsymbol{s}^s \boldsymbol{t}^t$$

for $k \in 0..n$.

To determine whether a run starts in a given genome, the previous genome is also needed. Thus, it is helpful to partition $P_k = W_k + X_k + Y_k + Z_k$ into the four possibilities that the last genome can take:

$$W_k := \sum_{a=0}^{k}\sum_{b=0}^{k}\sum_{c=0}^{k}\sum_{r=0}^{\lceil k/2\rceil}\sum_{s=0}^{\lceil k/2\rceil}\sum_{t=0}^{\lceil k/2\rceil} \Pr\begin{pmatrix} A_k = a, & B_k = b, & C_k = c, \\ R_k = r, & S_k = s, & T_k = t, \\ a_k = 0, & b_k = 0 \end{pmatrix} \cdot \boldsymbol{a}^a \boldsymbol{b}^b \boldsymbol{c}^c \boldsymbol{r}^r \boldsymbol{s}^s \boldsymbol{t}^t$$

$$X_k := \sum_{a=0}^{k}\sum_{b=0}^{k}\sum_{c=0}^{k}\sum_{r=0}^{\lceil k/2\rceil}\sum_{s=0}^{\lceil k/2\rceil}\sum_{t=0}^{\lceil k/2\rceil} \Pr\begin{pmatrix} A_k = a, & B_k = b, & C_k = c, \\ R_k = r, & S_k = s, & T_k = t, \\ a_k = 0, & b_k = 1 \end{pmatrix} \cdot \boldsymbol{a}^a \boldsymbol{b}^b \boldsymbol{c}^c \boldsymbol{r}^r \boldsymbol{s}^s \boldsymbol{t}^t$$

$$Y_k := \sum_{a=0}^{k}\sum_{b=0}^{k}\sum_{c=0}^{k}\sum_{r=0}^{\lceil k/2\rceil}\sum_{s=0}^{\lceil k/2\rceil}\sum_{t=0}^{\lceil k/2\rceil} \Pr\begin{pmatrix} A_k = a, & B_k = b, & C_k = c, \\ R_k = r, & S_k = s, & T_k = t, \\ a_k = 1, & b_k = 0 \end{pmatrix} \cdot \boldsymbol{a}^a \boldsymbol{b}^b \boldsymbol{c}^c \boldsymbol{r}^r \boldsymbol{s}^s \boldsymbol{t}^t$$

$$Z_k := \sum_{a=0}^{k}\sum_{b=0}^{k}\sum_{c=0}^{k}\sum_{r=0}^{\lceil k/2\rceil}\sum_{s=0}^{\lceil k/2\rceil}\sum_{t=0}^{\lceil k/2\rceil} \Pr\begin{pmatrix} A_k = a, & B_k = b, & C_k = c, \\ R_k = r, & S_k = s, & T_k = t, \\ a_k = 1, & b_k = 1 \end{pmatrix} \cdot \boldsymbol{a}^a \boldsymbol{b}^b \boldsymbol{c}^c \boldsymbol{r}^r \boldsymbol{s}^s \boldsymbol{t}^t.$$

Using the initial conditions $W_0 = 1$, $X_0 = Y_0 = Z_0 = 0$ and the system of recurrence relations below for successive $i \in 1..n$, one can inductively compute $P_0$, $P_1$, $P_2$, $P_3$, $\ldots$ and finally $P_n$.

| profile of the two genes at genome $i$ | | profile of the two genes at genome $i-1$ | | | | |
|---|---|---|---|---|---|---|
| | | type $W$<br>$a_{i-1}b_{i-1}$ = 00<br>($c_{i-1}$ = 0) | type $X$<br>$a_{i-1}b_{i-1}$ = 01<br>($c_{i-1}$ = 0) | type $Y$<br>$a_{i-1}b_{i-1}$ = 10<br>($c_{i-1}$ = 0) | type $Z$<br>$a_{i-1}b_{i-1}$ = 11<br>($c_{i-1}$ = 1) | |
| type $W$<br>$a_ib_i$ = 00<br>($c_i$ = 0) | | 00 00 00 $r_is_it_i$ = 000 | 00 10 00 $r_is_it_i$ = 000 | 10 00 00 $r_is_it_i$ = 000 | 10 10 10 $r_is_it_i$ = 000 | |
| | $(1-w_i)^2 \cdot [$ | $W_{i-1}$ | $+\ X_{i-1}$ | $+\ Y_{i-1}$ | $+\ Z_{i-1}$ | $] = W_i$ |
| type $X$<br>$a_ib_i$ = 01<br>($c_i$ = 0) | | 00 01 00 $r_is_it_i$ = 010 | 00 11 00 $r_is_it_i$ = 000 | 10 01 00 $r_is_it_i$ = 010 | 10 11 10 $r_is_it_i$ = 000 | |
| | $(1-w_i)w_i\boldsymbol{b} \cdot [$ | $W_{i-1}\boldsymbol{s}$ | $+\ X_{i-1}$ | $+\ Y_{i-1}\boldsymbol{s}$ | $+\ Z_{i-1}$ | $] = X_i$ |
| type $Y$<br>$a_ib_i$ = 10<br>($c_i$ = 0) | | 01 00 00 $r_is_it_i$ = 100 | 01 10 00 $r_is_it_i$ = 100 | 11 00 00 $r_is_it_i$ = 000 | 11 10 10 $r_is_it_i$ = 000 | |
| | $w_i(1-w_i)\boldsymbol{a} \cdot [$ | $W_{i-1}\boldsymbol{r}$ | $+\ X_{i-1}\boldsymbol{r}$ | $+\ Y_{i-1}$ | $+\ Z_{i-1}$ | $] = Y_i$ |
| type $Z$<br>$a_ib_i$ = 11<br>($c_i$ = 1) | | 01 01 01 $r_is_it_i$ = 111 | 01 11 01 $r_is_it_i$ = 101 | 11 01 01 $r_is_it_i$ = 011 | 11 11 11 $r_is_it_i$ = 000 | |
| | $w_i^2\boldsymbol{abc} \cdot [$ | $W_{i-1}\boldsymbol{rst}$ | $+\ X_{i-1}\boldsymbol{rt}$ | $+\ Y_{i-1}\boldsymbol{st}$ | $+\ Z_{i-1}$ | $] = Z_i$ |

For conditional probabilities that do not mention all six variables, it is easy to streamline the recurrence relations to enable more efficient calculations. One need merely substitute 1 for undesired variables and simplify. For example, for weighted hypergeometric $p$-values $\Pr(C_n \geq c \mid A_n = a, B_n = b)$ we can substitute $\boldsymbol{r} \leftarrow 1$, $\boldsymbol{s} \leftarrow 1$, and $\boldsymbol{t} \leftarrow 1$ to obtain the reduced system

$$
\begin{aligned}
W'_0 &= 1 \\
X'_0 &= Y'_0 = Z'_0 = 0 \\
W'_i &= (1-w_i)^2 \cdot \big(W'_{i-1} + X'_{i-1} + Y'_{i-1} + Z'_{i-1}\big) \\
X'_i &= (1-w_i)w_i\boldsymbol{b} \cdot \big(W'_{i-1} + X'_{i-1} + Y'_{i-1} + Z'_{i-1}\big) \\
Y'_i &= w_i(1-w_i)\boldsymbol{a} \cdot \big(W'_{i-1} + X'_{i-1} + Y'_{i-1} + Z'_{i-1}\big) \\
Z'_i &= w_i^2\boldsymbol{abc} \cdot \big(W'_{i-1} + X'_{i-1} + Y'_{i-1} + Z'_{i-1}\big) \\
P'_i &= W'_i + X'_i + Y'_i + Z'_i
\end{aligned}
$$

which simplifies to

$$
\begin{aligned}
P'_0 &= 1 \\
P'_i &= \big((1-w_i)^2 + (1-w_i)w_i\boldsymbol{b} + w_i(1-w_i)\boldsymbol{a} + w_i^2\boldsymbol{abc}\big) \cdot P'_{i-1}
\end{aligned}
\tag{†}
$$

so that $\Pr(A_n = a, B_n = b, C_n = c)$ is the coefficient $[\boldsymbol{a}^a\boldsymbol{b}^b\boldsymbol{c}^c]P'_n$ of $\boldsymbol{a}^a\boldsymbol{b}^b\boldsymbol{c}^c$ in $P'_n$ and

$$
\Pr(C_n \geq c \mid A_n = a, B_n = b) = \frac{\sum_{c'=c}^{n} \Pr(A_n = a, B_n = b, C_n = c')}{\sum_{c'=0}^{n} \Pr(A_n = a, B_n = b, C_n = c')} = \frac{\sum_{c'=c}^{n} [\boldsymbol{a}^a\boldsymbol{b}^b\boldsymbol{c}^{c'}]P'_n}{\sum_{c'=0}^{n} [\boldsymbol{a}^a\boldsymbol{b}^b\boldsymbol{c}^{c'}]P'_n}.
$$

We remark that when all the weights are the same, i.e., $w_i = w'$ for all $i \in 1..n$ for any constant $w' \in (0,1)$, weighted hypergeometric $p$-values reduce to the original unweighted hypergeometric $p$-values.

For weighted runs $p$-values $\Pr(T_n \le t \mid C_n = c)$, we substitute $\boldsymbol{a} \leftarrow 1$, $\boldsymbol{b} \leftarrow 1$, $\boldsymbol{r} \leftarrow 1$, and $\boldsymbol{s} \leftarrow 1$ to obtain the reduced system

$$\begin{aligned}
W_0'' &= 1\\
X_0'' &= Y_0'' = Z_0'' = 0\\
W_i'' &= (1-w_i)^2 \quad \cdot \left(W_{i-1}'' + X_{i-1}'' + Y_{i-1}'' + Z_{i-1}''\right)\\
X_i'' &= (1-w_i)w_i \cdot \left(W_{i-1}'' + X_{i-1}'' + Y_{i-1}'' + Z_{i-1}''\right)\\
Y_i'' &= w_i(1-w_i) \cdot \left(W_{i-1}'' + X_{i-1}'' + Y_{i-1}'' + Z_{i-1}''\right)\\
Z_i'' &= w_i^2\boldsymbol{c} \cdot \left(W_{i-1}''\boldsymbol{t} + X_{i-1}''\boldsymbol{t} + Y_{i-1}''\boldsymbol{t} + Z_{i-1}''\right)\\
P_i'' &= W_i'' + X_i'' + Y_i'' + Z_i''
\end{aligned}$$

which simplifies under $Q_i'' := W_i'' + X_i'' + Y_i''$ to

$$\begin{aligned}
Q_0'' &= 1\\
Z_0'' &= 0\\
Q_i'' &= (1-w_i^2) \cdot \left(Q_{i-1}'' + Z_{i-1}''\right)\\
Z_i'' &= w_i^2\boldsymbol{c} \cdot \left(Q_{i-1}''\boldsymbol{t} + Z_{i-1}''\right)\\
P_i'' &= Q_i'' + Z_i''
\end{aligned} \tag{‡}$$

so that $\Pr(C_n = c, T_n = t) = [\boldsymbol{c}^c\boldsymbol{t}^t]P_n''$ and

$$\Pr(T_n \le t \mid C_n = c) = \frac{\sum_{t'=0}^{t} \Pr(C_n = c, T_n = t')}{\sum_{t'=0}^{\lceil n/2 \rceil} \Pr(C_n = c, T_n = t')} = \frac{\sum_{t'=0}^{t} [\boldsymbol{c}^c\boldsymbol{t}^{t'}]P_n''}{\sum_{t'=0}^{\lceil n/2 \rceil} [\boldsymbol{c}^c\boldsymbol{t}^{t'}]P_n''}.$$

Derivations of recurrences customized for any others that are desired, e.g., $\Pr(C_n \le c \mid T_n = t)$, $\Pr(C_n \ge c \mid R_n = r, S_n = s)$, $\Pr(C_n \ge c)$, or $\Pr(T_n \le t \mid A_n = a, B_n = b)$, proceed similarly.

### Calculation of $p$-values

The easiest implementations use full multi-dimensional rectangular arrays of IEEE 754 double-precision floating-point values. For example, consider (†). Suppose we store a polynomial in $\boldsymbol{a}$, $\boldsymbol{b}$, $\boldsymbol{c}$ as a cubical array with increasing successive powers 0, 1, 2, … of $\boldsymbol{a}$ going front-to-back, of $\boldsymbol{b}$ going top-to-bottom, and of $\boldsymbol{c}$ going left-to-right. We start with an array consisting of a single element +1.0, i.e., $P_0'$.

For successive $i \in 1..n$, replace the array with the entrywise sum of four arrays (corresponding to the four terms of the first factor of the right-hand side of $P_i'$): (1) the current array with every entry multiplied by $(1-w_i)^2$ and padded by a 1-entry-thick slab of +0.0's on the back, bottom, and right; (2) the current array with every entry multiplied by $(1-w_i)w_i$ and padded by a 1-entry-thick slab of +0.0's on the back, top, and right; (3) the current array with every entry multiplied by $w_i(1-w_i)$ and padded by a 1-entry-thick slab of +0.0's on the front, bottom, and right; and (4) the current array with every entry multiplied by $w_i^2$ and padded by a 1-entry-thick slab of +0.0's on the front, top, and left. The final array gives $P_n'$ and is easily post-processed in a single last pass to obtain a full set of the desired $p$-values. Time used is $\Theta(n^4)$ and space used is $\Theta(n^3)$ (with small hidden constants). Scoring of a particular gene pair reduces to a single array lookup.

As another example, consider (‡). We might choose to store a polynomial in $\boldsymbol{c}$ and $\boldsymbol{t}$ as a rectangular array with increasing successive powers 0, 1, 2, … of $\boldsymbol{c}$ going top-to-bottom and of $\boldsymbol{t}$ going left-to-right. We start with two arrays, each consisting of a single element: `Q` with +1.0 and `Z` with +0.0.

For successive $i \in 1..n$, simultaneously update `Q` and `Z` as follows: replace `Q` with the entrywise sum of the two current arrays after multiplying each element by $1 - w_i^2$ and padding by a single row and column of +0.0's on the bottom and right, and replace `Z` by the following: take the entrywise sum of the current `Q` after padding by a single row and column of +0.0's on the top and left with the current `Z` after padding by a single row and column of +0.0's on the top and right, then multiply every entry by $w_i^2$. Take the entrywise

sum of the final `Q` and `Z` arrays to obtain $P''_n$, which is easily post-processed in a single last pass to obtain a full set of the desired $p$-values. Time used is $\Theta(n^3)$ and space used is $\Theta(n^2)$ (with small hidden constants). Again, scoring of a particular gene pair reduces to a single array access.

Many refinements are possible, although not required for a problem as small as $n = 214$. For example, by updating coefficients in a reverse lexicographic monomial order, one can work completely in-place and thus limit storage usage to the size of the final answers plus a very small constant number of temporary storage locations; no temporary arrays are needed.

Further, when full rectangular multi-dimensional arrays are used, many of the entries will be necessarily zero and do not need to be stored. To illustrate, $C_k$ actually takes on a more restricted range than $0..k$; in fact, $C_k \in \max(0, A_k + B_k - n)..\min(A_k, B_k)$. 2 GiB of memory holds 268,435,456 IEEE 754 doubles. As $(n+1)^3$ last stays within this at $n = 644$, this is the limit for in-memory computation of weighted hypergeometric $p$-values with full rectangular arrays. If one stores only the more restricted range of $C_k$, however, $n = 1{,}170$ is the in-memory computation limit (when rectangular arrays would be using more than 11.9 GiB). Runs obey more complicated bounds that are not detailed here, but as only two variables are needed for our weighted runs $p$-values, the order of growth of space requirements for computing them is much less and perfect space-efficiency in computing them is less critical.

The simple access patterns demanded of the coefficients make streaming coefficients from disk storage or communicating slivers of arrays across machines in a parallel-computing cluster relatively easy should this be required. Also, with 64-bit computing and large amounts of silicon memory becoming commonplace, 2 GiB is not the barrier it once was and computing $p$-values for thousands for genomes is not expected to pose undue difficulty.

A last computational point that must be addressed is numerical accuracy. Note that the probability of observing a particular pair of gene profiles (an "elementary event") is

$$\prod_{i=1}^{n} \begin{pmatrix} w_i & \text{if } a_i = 1 \\ 1 - w_i & \text{if } a_i = 0 \end{pmatrix} \begin{pmatrix} w_i & \text{if } b_i = 1 \\ 1 - w_i & \text{if } b_i = 0 \end{pmatrix} \geq \prod_{i=1}^{n} \min(w_i, 1 - w_i)^2,$$

which with the particular profiles used here is above $10^{-242}$. This is well above IEEE 754 double's lower limit of approximately $10^{-300}$. As all events are disjoint unions of these elementary events, no event has a probability that is troublesome to represent. Further, as all quantities in our calculations are positive and our chains of operations are relatively short (and involve no subtractions), we expect only the last few bits of our $p$-values to be incorrect and to have near-full (e.g., 10+ decimal digits) accuracy. As $n$ gets larger or $w_i$ get closer to 0 or 1, the probability of observing particular profiles may become so small that they underflow to zero in IEEE 754 double precision. One may then need to switch representations (e.g., separating exponents) or switch to software-based arbitrary-precision floating-point (e.g., as implemented in packages such as GNU MP, *Mathematica*, and *Maple*).

Note that in our case there are $2^{214} \cdot 2^{214} = 2^{428}$ elementary events. Hence, brute-force enumeration of elementary events and adding up their individual probabilities is an infeasible approach to computation of the $p$-values we require.
