# Supplementary material for: An improved method for identifying functionally linked proteins using phylogenetic profiles
Source: BMC Bioinformatics. 2007 May 22;8(Suppl 4):S7. doi: 10.1186/1471-2105-8-S4-S7 (PMC1892086; doi:10.1186/1471-2105-8-S4-S7)
Supplement: Additional file 2 — Distance matrix before and after optimal swivelling. This one-page PDF file shows the hierarchically-clustered-by-complete-linkage genome-genome Jaccard dissimilarity distance matrix before (left) and after (right) optimal swivelling. The improved visual appearance of the swivelled distance matrix is apparent. The effect can be even more dramatic when optimal swivelling is applied to heatmaps of, e.g., microarray expression data. [file 1471-2105-8-S4-S7-S2.pdf]

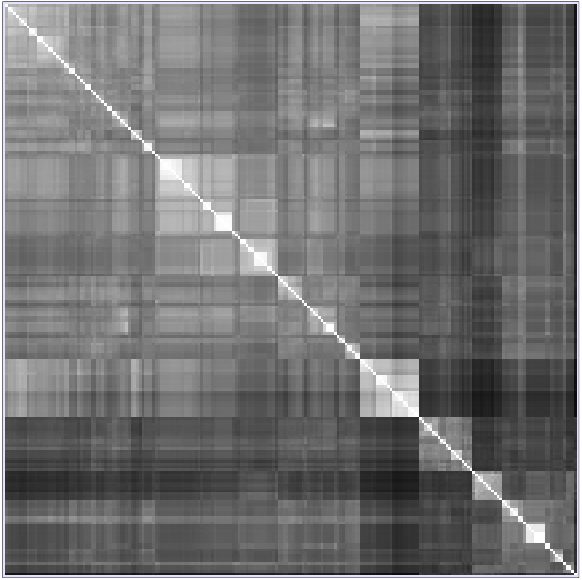

Jaccard organism-organism distance matrix just after *Mathematica* hierarchical clustering with complete linkage

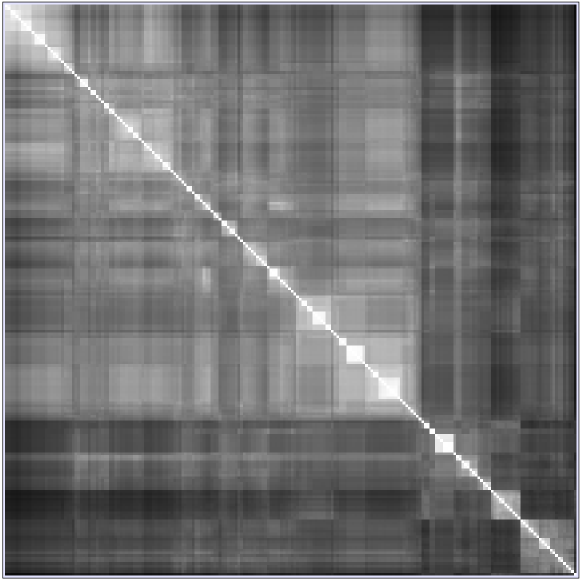

Jaccard organism-organism distance matrix after hierarchical clustering with complete linkage and optimal swivelling to minimize sum of squares of Jaccard dissimilarities of adjacent organisms
