# Supplementary figures and images for: An improved method for identifying functionally linked proteins using phylogenetic profiles
Source: BMC Bioinformatics. 2007 May 22;8(Suppl 4):S7. doi: 10.1186/1471-2105-8-S4-S7 (PMC1892086; doi:10.1186/1471-2105-8-S4-S7)

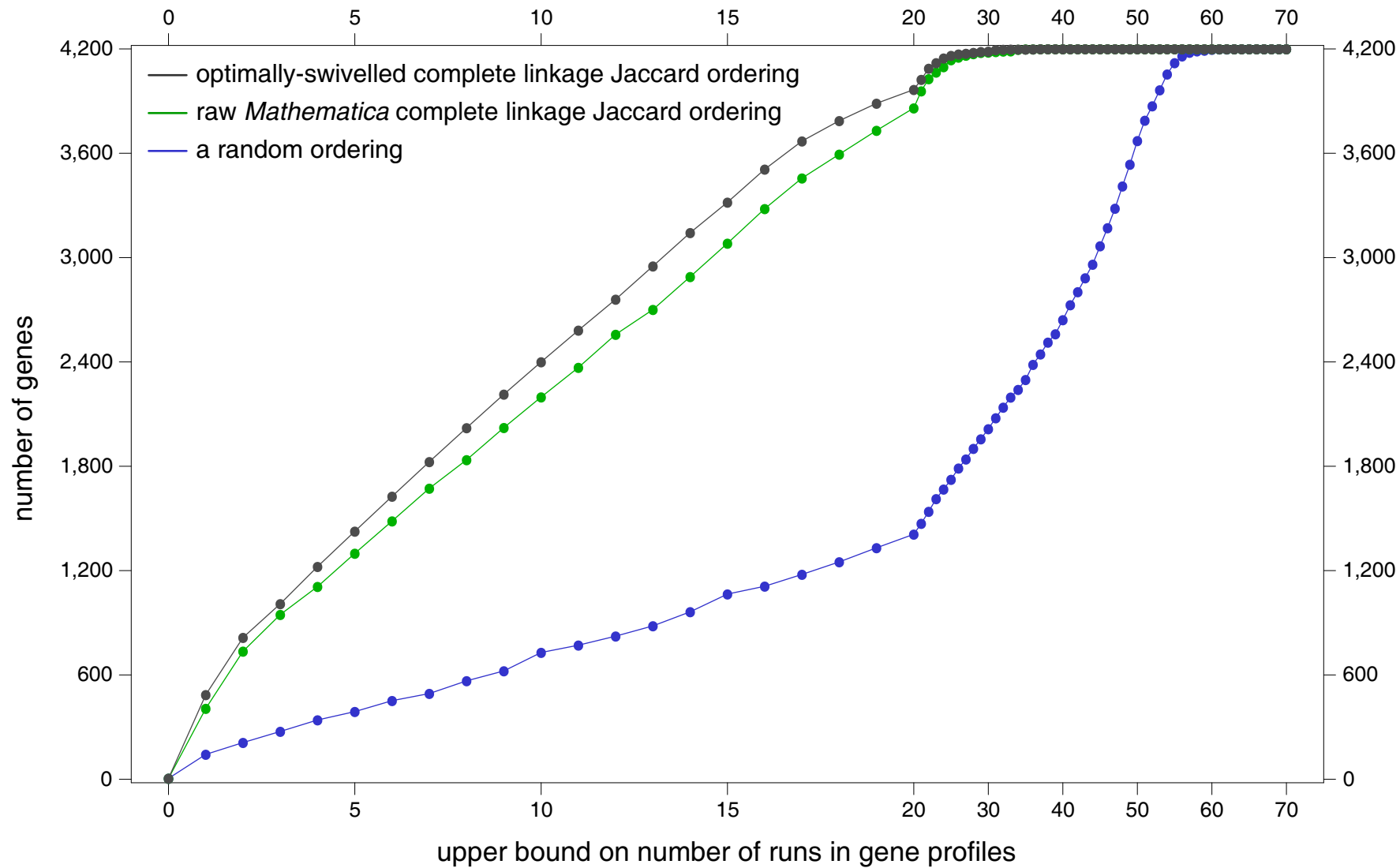

Supplement: Additional file 3 — Reduction in the number of runs per gene after optimal swivelling. This one-page PDF file shows the cumulative number of genes as the number of runs in the gene's profile is slowly raised. It is apparent that optimal swivelling tends to reduce the number of runs in a gene's profile. Thus, the organism order derived from optimal swivelling captures the organisms' underlying phylogeny better than the order derived from hierarchical clustering without optimal swivelling (which, in turn, does much better than a random ordering, suggesting that runs can indeed capture phylogenetic information). [file 1471-2105-8-S4-S7-S3.pdf]
